# Nanotechnology for Environmental Remediation: Materials and Applications

**DOI:** 10.3390/molecules23071760

**Published:** 2018-07-18

**Authors:** Fernanda D. Guerra, Mohamed F. Attia, Daniel C. Whitehead, Frank Alexis

**Affiliations:** 1 Department of Bioengineering, Clemson University, 301 Rhodes Research Center, Clemson, SC 29634, USA; fdlbqgrr@memphis.edu (F.D.G.); mattia@clemson.edu (M.F.A.); 2 Department of Chemistry, Clemson University, 467 Hunter Laboratories, Clemson, SC 29634, USA; 3 School of Biological Sciences and Engineering, Yachay Tech, San Miguel de Urcuquí, Ibarra EC 100150, Ecuador

**Keywords:** nanotechnology, nanomaterials, environmental remediation, nanostructures, contaminants, pollutants

## Abstract

Environmental remediation relies mainly on using various technologies (e.g., adsorption, absorption, chemical reactions, photocatalysis, and filtration) for the removal of contaminants from different environmental media (e.g., soil, water, and air). The enhanced properties and effectiveness of nanotechnology-based materials makes them particularly suitable for such processes given that they have a high surface area-to-volume ratio, which often results in higher reactivity. This review provides an overview of three main categories of nanomaterials (inorganic, carbon-based, and polymeric-based materials) used for environmental remediation. The use of these nanomaterials for the remediation of different environmental contaminants—such as heavy metals, dyes, chlorinated organic compounds, organophosphorus compounds, volatile organic compounds, and halogenated herbicides—is reviewed. Various recent examples are extensively highlighted focusing on the materials and their applications.

## 1. Introduction

Environmental pollution is undoubtedly one of the main problems that society faces today. New technologies are constantly being explored for the remediation of contaminants of the air, water, and soil [1]. Particulate matter, heavy metals, pesticides, herbicides, fertilizers, oil spills, toxic gases, industrial effluents, sewage, and organic compounds are just a few examples of the many concerning contaminants [2,3]. Different types of materials can be employed in environmental remediation and therefore a wide variety of approaches can be exploited for this purpose. As the capture and degradation of environmental pollutants can be challenging due to the complexity of the mixture of different compounds, high volatility, and low reactivity; recent studies have focused on the use of nanomaterials for the development of new environmental remediation technologies [4].

Nanotechnology has gained a lot of attention in the past decades due to the unique physical properties of nanoscale materials. Nanomaterials present enhanced reactivity and thus better effectiveness when compared to their bulkier counterparts due to their higher surface-to volume ratio. In addition, nanomaterials offer the potential to leverage unique surface chemistry as compared to traditional approaches, such that they can be functionalized or grafted with functional groups that can target specific molecules of interest (pollutants) for efficient remediation. Further, the intentional tuning of the physical properties of the nanomaterials (such as size, morphology, porosity, and chemical composition) can confer additional advantageous characteristics that directly affect the performance of the material for contaminant remediation. The rich surface modification chemistry along with the tunable physical parameters of the nanomaterial offer significant advantages over conventional methods for addressing environmental contamination. By extension, methods that are developed as a combination of several different materials (hybrids/composites), gathering specific desired properties from each of its components, are potentially more efficient, selective, and stable than methods based upon a single nanoplatform. For instance, adhering nanoparticles to a scaffold can be an alternative way to increase the stability of the material when compared to the use of nanoparticles alone. Functionalizing material with specific chemicals responsible for targeting contaminant molecules of interest can help increase the selectivity and efficiency of the material [5,6,7,8,9].

It is important that the materials used for the remediation of pollution are not another pollutant themselves after they have been employed. Therefore, biodegradable materials are extremely interesting for this field of application. The use of biodegradable materials may not only increase consumer confidence and acceptance of a particular technology, in the sense that there is no generation of a material waste to be disposed of after the treatment, but it also could offer a greener and safer alternative for the environmental remediation of pollutants. Furthermore, new technologies that can rely on the target-specific capture of contaminants are especially attractive, as they can overcome low efficiencies derived from off-targeting. Therefore, different studies have focused on using the principles of nanotechnology and combining it with chemical and physical modification of the surface of the materials in an effort to obtain engineered materials that can overcome many of the challenges involved with the remediation of contaminants [10,11]. Target-specific capture, cost effectiveness, facile synthesis, green chemistry, non-toxicity, biodegradability, recyclability, and the potential for recovery after use (regeneration) are some of the key challenges that must be considered when developing new nanomaterials for environmental remediation. Despite the potential advantages of the nanomaterials mentioned above, some are inherently unstable under normal conditions, therefore their preparation requires special techniques for formulation at the nanoscale. Additional operations are needed to prevent agglomeration, enhance monodispersity, and increase stability. The possible toxicity of metallic nanoparticles involved in the remediation process, along with their byproducts and recovery costs from the remediation site is another factor that may impose a limitation to their use. This is why a keen understanding of the material platforms, their fabrication process, and performance optimization are required in order to develop good nanomaterial candidates capable of addressing environmental problems.

**Figure 1:**
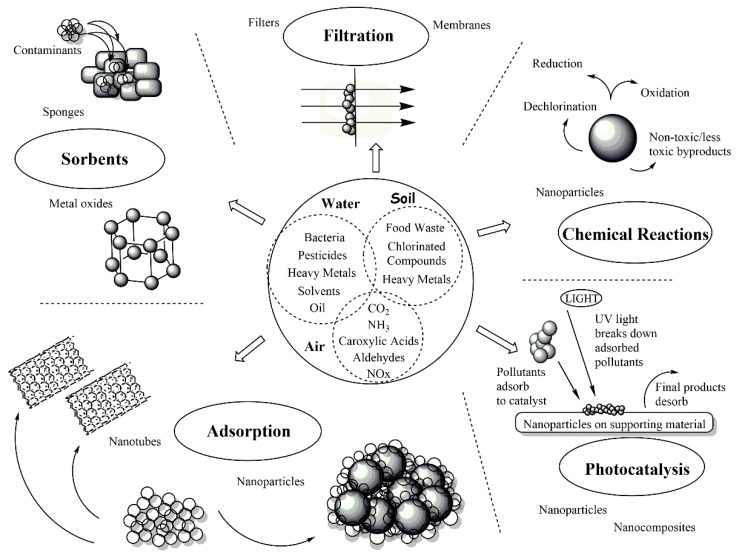
Environmental remediation approaches.

The goal of this review is to provide a general overview of some of the recent advances in the development of functional nanomaterials and nanocomposites used for the environmental remediation of a variety of pollutants. Among the many possible ways through which a contaminant can be remediated are absorption, adsorption, chemical reactions, photocatalysis, and filtration, as summarized in Figure 1 [1,12,13,14,15,16,17].

A variety of different materials can be used for the approaches described in Figure 1. To the best of our knowledge, there is no established classification regarding the different types of materials that can be employed for environmental remediation. Therefore, this review focuses on three main types of nanomaterials described in the literature: inorganic, carbon-based, and polymer-based materials. Each of these classes and their applications will be discussed in the following sections.

## 2. Inorganic Nanomaterials

### 2.1. Metal- and Metal Oxide-Based Nanomaterials

Different metal-based nanomaterials have been described for the remediation of numerous contaminants, but the vast majority of studies have been dedicated to the removal of heavy metals and chlorinated organic pollutants from water. Metal and metal oxide nanomaterials are highly efficient adsorbents exhibiting advantages such as fast kinetics and high adsorption capacity [18]. Nanoparticles are commonly used for environmental remediation, since they are highly flexible towards both in situ and ex situ applications in aqueous systems [19]. Table 1 summarizes some of the different metal-based materials that have been investigated for different environmental remediation applications.

Efficient synthetic methods to obtain shape-controlled, very stable, and monodisperse metal/metal oxide nanomaterials have been extensively investigated during the last decade following both physical and chemical approaches. Among these synthetic protocols, thermal decomposition and/or reduction, co-precipitation [54,55], and hydrothermal synthetic protocols are widely used and are easily scalable with high yields.

Silver nanoparticles (AgNPs) are well known for their significant antibacterial, antifungal, and antiviral activity, and thus applied as water disinfectants [20,21,22]. For instance, AgNPs of less than 10 nm in diameter were found to be highly toxic to *Escherichia coli* and *Pseudomonas aeruginosa*. They can also prevent viruses from binding to host cells by preferentially binding to the virus’ glycoproteins. Slightly larger particle sizes (i.e., 11–23 nm) afford lower bactericidal activity [56]. In addition, triangular AgNPs exhibited better antibacterial effects than Ag nanorods and Ag nanospheres, emphasizing the importance of not only size, but also shape of the particles in eliciting their desired effects [24]. AgNPs have been coupled with several other materials such as metal oxides and polymers with the goal of improving the overall efficiency of the resulting nanocomposite, as will be discussed later.

Another frequently investigated metal-based material for environmental remediation is titanium oxides. TiO_2_ NPs have been extensively studied for waste treatment, air purification, self-cleaning of surfaces, and as a photocatalyst in water treatment application due to their characterized low-cost, nontoxicity, semiconducting, photocatalytic, electronic, gas sensing, and energy converting properties [57,58]. TiO_2_ NPs are activated by light, and thus frequently studied for their ability to remove organic contaminants from various media. TiO_2_ NPs are capable of producing highly reactive oxidants like hydroxyl radicals that serve as a disinfectant for microorganisms such as fungi, bacteria, viruses, and algae [25,59]. Since TiO_2_ exhibits a rather limited photocatalytic capability, the material is typically doped with another transition metal ion to increase performance. Therefore, several studies have investigated metal-doped TiO_2_ NPs. Park and Lee [30], described the synthesis of TiO_2_ nanofibers (control) and Ag-doped TiO_2_ nanofibers by a sol–gel electrospinning technique. These materials were then evaluated as photocatalyst candidates for the photocatalytic degradation of 2-chlorophenol under UV irradiation. Ag-doped TiO_2_ nanofibers presented increased photodegradation when compared to the TiO_2_ nanofibers. The increase was attributed to four possible factors, including, adequate amount of Ag on the surface that effectively captured photoinduced electrons and holes, quick transfer of photoinduced electrons to the adsorbed oxygen present on the surface of the nanofibers, increased amount of surface hydroxyl groups, and expanded response range to light to the visible region.

In addition to titanium oxide materials, titanates (i.e., inorganic compounds of titanium oxide) have also been reported for the removal of contaminants. For instance, Chen et al. [35], reported the fabrication of basic, acidic, and neutral titanate nanotubes (TNTs) by a hydrothermal method, and these materials were evaluated for the catalytic reduction of NO with ammonia. Manganese oxides were loaded to the three TNT formulations to yield Mn-doped titanate nanosheets, titanate nanorods, and titanate nanotubes (i.e., Mn/TNTs) in the case of basic, acidic, and neutral pH media, respectively. The results showed that the neutral Mn/TNTs exhibited the largest surface area, the best dispersion of active species, and the most active redox performance of the series. Thus the neutral Mn/TNTs had the best catalytic reduction activity, whereas the basic Mn/TNTs showed negligible activity.

Moreover, mixed oxide materials have also been investigated. Rasalingam et al. [36] reported the preparation of TiO_2_-SiO_2_ binary mixed oxide materials using bamboo as a silica source and titanium isopropoxide or titanium butoxide. The materials were evaluated for the photocatalytic degradation of methylene blue dye. The results showed a significant photoactivity as indicated by the degradation rate of methylene blue at varied treatment times. They suggested that the composite may have potential applications in smaller-scale industrial wastewater treatment systems. These mixed oxide materials have enhanced abilities to remove a wide variety of pollutants. On balance, it has been pointed out that despite the fact that these binary mixed oxides show better activity than pure TiO_2_ materials in most instances, their utilization is limited for the mineralization of selected pollutants [36].

Furthermore, magnetic metallic nanoadsorbents are particularly attractive as they can be easily retained and separated from treated water. Iron and iron oxides NPs are extensively described in the literature for the removal of different heavy metals—such as Ni^2+^ [37,38], Cu^2+^ [39], Co^2+^ [37], and Cd^2+^ [38]—as well as for the remediation of chlorinated organic solvents [40,41]. Nonetheless, there are some challenges when using this class of NPs for the remediation of environmental contaminants. Aggregation is one of the major concerns as it can significantly affect the reactivity of the material, and consequently reduce the advantage of using nanoscale materials as a means of improving efficiency. Another challenge when working with metal and metal oxide NPs is due to the possible toxicity of the materials involved. In addition, the associated cost and fate of the remediation technology are also important factors to consider when opting for the use of NPs as a remediation material. Some examples of strategies for overcoming these challenges are presented in this section.

**Figure 2:**
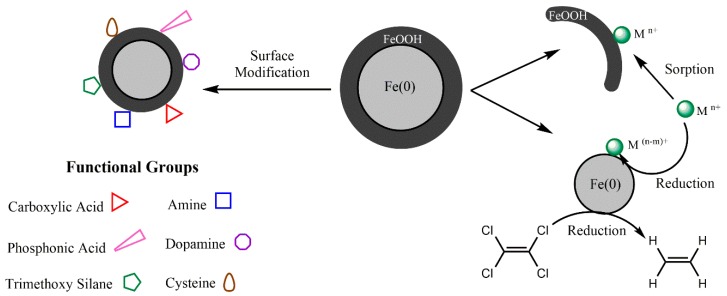
Degradation mechanism of chlorinated contaminants and heavy metals from aqueous systems using iron NPs.

Iron NPs typically exhibit a core–shell structure, with elemental iron (i.e., Fe^0^, also termed “zerovalent”) comprising the core and mixed valent (Fe(II) and Fe(III)) oxides forming the shell [60]. Figure 2 illustrates the mechanisms through which iron NPs can be used for the remediation of environmental contaminants. Both chlorinated compounds and heavy metals can be reduced via electron donation from the zerovalent iron core. Additionally, the shell portion of the NPs can also facilitate the remediation of contaminants, such as those heavy metals that present a higher standard reduction potential (E^0^) than of the Fe^2+^/Fe couple [42].

In an effort to investigate how to overcome aggregation of the NPs, Hooshyar explored the use of sonication of the iron NP solution to enhance Ni^2+^ and Co^2+^ removal [37]. It has been demonstrated that nanoclusters can be dispersed when exposed to sonication, but the freed NPs are subject to re-aggregation if submitted to longer sonication times. The iron NPs used in the experiments were spherical and 12 nm in diameter. Hooshyar and co-workers [37] demonstrated that sonication of the iron nanoparticle solution can enhance the removal of Ni^2+^ and Co^2+^ with an optimal sonication time of approximately 20 min for nickel removal and 30 min for cobalt removal. The maximum removal efficiencies obtained for each case were 38% for nickel and 59% for cobalt.

Several studies have investigated the use of bimetallic NPs as a means of overcoming some of the limitations associated with monometallic NPs including their propensity toward aggregation and their low stability. Often, different stabilizers and surfactants are employed to increase the stability of nanoparticle solutions, however, the addition of a second metal to the formulation can enhance the solution stability of the material and obviate the need for stabilizers and surfactants [61]. Improved stability can contribute to increased efficiency and capacity, and can accelerate the degradation rate of contaminants [62]. The incorporation of a second metal—such as Pd [44,46,47,48,63], Ni [49,50,51,63], or Cu [52,53]—has been reported in the literature as a strategy for enhancing the stability of zero-valent iron NPs (nZVI). Some noble metals (i.e., resistant to corrosion and oxidation in moist air) can be combined with nZVI to catalyze dechlorination and hydrogenation reactions with contaminants, therefore resulting in a more efficient remediation method [64]. For instance, in their study of the effects of the addition of Pd to nZVI particles, Wang et al. described an enhanced rate of dehalogenation of chlorinated organic compounds, albeit at a higher cost due to the expense of elemental palladium [48]. Other alternatives to enhance the stability of NPs include the use of supporting materials, such as several examples that follow in our discussion of polymer-based materials (vide infra).

Furthermore, another concern regarding the use of metal-based NPs is due to the possible toxicity of the chemicals used to synthesize the material and of the byproducts generated from the contaminant degradation. Poguberovic et al. successfully demonstrated the use of nZVI for the removal of Ni^2+^ and Cu^2+^ from aqueous solutions [39]. The nZVI used in the experiments were synthesized using oak and mulberry leaf extracts. The compounds present in these high antioxidant extracts react with iron (III) to form nZVI [65]. Using natural products for the fabrication of environmental remediation materials is important to overcome concerns regarding the possible toxicity of chemicals and byproducts when using chemical synthesis approaches. In addition, the “green” synthesis of nZVI demonstrated by Poguberovi and co-workers has the advantages of adding value to natural resources, such as the leaf extracts, that are otherwise considered waste and providing a low-cost adsorbent for the remediation of heavy metals from water. The study demonstrates fast kinetics and rate of adsorption of the nZVI, with adsorption isotherm results showing a higher capacity for Ni^2+^, 777.3 mg Ni/g when using oak leaf extracts, and higher capacity for Cu^2+^, 1047 mg Cu/g when using mulberry leaf extracts, highlighting that interesting differences in performance can arise from the particular raw material used for fabrication. The maximum removal of Ni^2+^ was achieved at pH = 8.0, while the maximum removal of Cu^2+^ was achieved at pH = 7.0. While this study is promising, further investigation is necessary for a full-scale application of this material in wastewater treatment. Furthermore, a pH sensitive remediation technology may impose limitations to its applicability to in situ remediation, as some of the environmental scenarios may not present adequate conditions for the remediation to occur successfully and efficiently.

Moreover, a binary mixed magnetic nanoparticle system has been developed by Ding et al. [66]. They synthesized lecithin-loaded Ni/Fe nanoparticles by microemulsion method and tested these materials for the remediation of 3,3′,4,4′-tetrachlorobiphenyl (PCB77) as a target pollutant. Lecithin was used as a biocompatible surfactant for forming monodispersed and stable lecithin-Ni/Fe NPs and to capture the targeted organic contaminant. The results revealed high efficiency and rapid removal of PCB77 by lecithin-nano Ni/Fe as compared to a control carrier (i.e., unmodified nanolecithin). Thus, they succeeded in the development of a relatively nontoxic and inexpensive organic–inorganic combined material for targeting polychlorinated biphenyl contaminants.

A recent study has used five plant extracts and juices for the production of nanoiron suspensions and their application for Cr^6+^ removal [43]. Due to the antioxidant effect of polyphenols present in extracts, they are employed as reducing agents for iron ions in aqueous solutions which led to producing nanoiron. The maximum extraction of polyphenols from herbs was achieved at an herb mass to water ratio ranging from 10 to 20 g/L. The percentage of nanoiron to total iron in the initial solution did not exceed 50%. The maximum and the minimum concentrations of the iron NPs formed in the suspensions was 1.2 g/L (22 mM) and 1 g/L (18 mM) respectively. It was found that they were effective for Cr^6+^ removal, reaching reduction rates as high as 500 mg Cr^6+^ per g of iron NPs, which is significantly higher than the values reported in the literature for both mZVI and nZVI.

Although a variety of materials have been described in the literature due to their potential application in environmental remediation, few studies have evaluated or reported the use of their technology on the field [67,68,69,70,71,72,73,74,75,76,77]. In this regard, NZVI materials have been extensively investigated due to their great reduction potential towards chlorinated contaminants. Su et al. [67] reported a field study on the performance of emulsified zero valent iron (EZVI) nanoparticles for the treatment of chlorinated volatile organic compounds (CVOCs) in a contaminated groundwater site. The test was conducted and monitored over a period of two and a half years, and the results demonstrated significant reductions in total CVOC mass. The mechanism of the apparent effective remediation was reported to be a direct abiotic dechlorination by nanoiron followed by biological reductive dechlorination stimulated by the corn oil present in the emulsion formulation. Decreased concentration of CVOCs was in accordance with observed increased concentration of degradation products, such as ethene. The study reports a total CVOC mass decrease of 86% by the end of the 2.5 years monitoring period. These findings suggest the application and functionality of EZVI nanoparticles for a successful in situ remediation but limited to chlorinated compounds.

In a similar manner, Mackenzie et al. [68] reported the use of Carbo-Iron, a composite material of colloidal activated carbon and embedded nanoiron, for the remediation of tetrachloroethene (PCE) in a contaminated field site in Germany. The study was conducted in two steps, with an initial smaller test injection of material, followed by a second, main injection, about 15 months after the first test injection. The authors reported similar profiles observed for both injections, with an initial high concentration of PCE, of approximately 20 mg/L, followed by a decrease soon after the particles were injected, and a slow reappearance of the contaminant after 2–3 months. For the first round of injections, the rebound of PCE concentration was attributed to the small amount of materials used. However, the authors did not explain why the same pattern was observed for the second round of injections. The concentration of PCE was determined to be 1.5 μg/L 200 days after the second injection, but levels of up to 8–9 mg/L were reported 1000 days after the injection. These results suggest an initial significantly successful application of the material for in situ remediation; however, the authors did not provide sufficient explanations to account for the elevation of PCE concentration 200 days after the second injection.

### 2.2. Silica Nanomaterials

Due to their versatility, mesoporous silica materials have gained attention for various applications, such as adsorption and catalysis. Mesoporous silica materials possess a number beneficial features for environmental remediation applications including: high surface area, facile surface modification, large pore volumes, and tunable pore size [78]. Due to their exquisite performance as adsorbents, a variety of studies have reported the use of these materials for contaminant remediation in the gas phase. Furthermore, different surface modifications of mesoporous silica materials have been reported in many publications [79,80,81,82,83,84,85,86,87,88,89,90]. Table 2 summarizes some of the reported works found in the literature that investigate the use of silica nanomaterials for environmental remediation of different contaminants.

**Figure 3:**
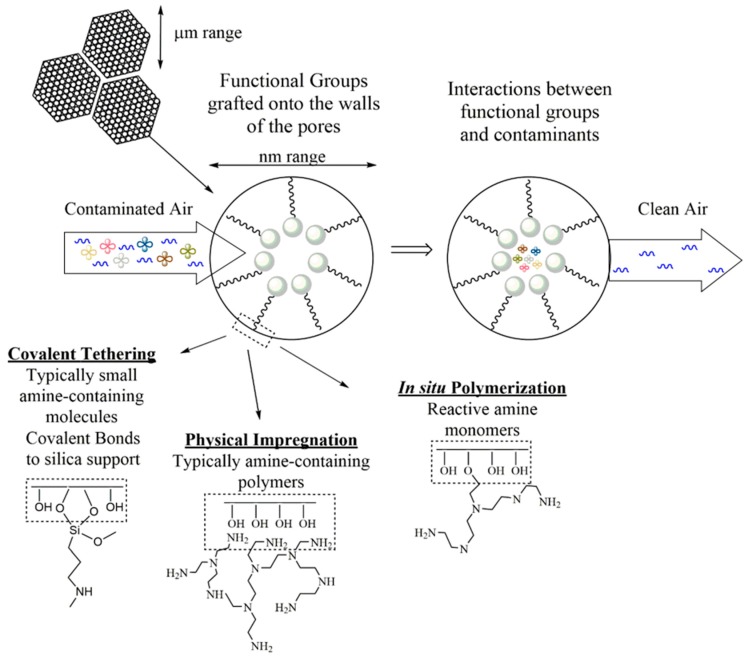
An example of mesoporous silica materials used for environmental remediation of contaminants.

The hydroxyl groups present on the surface of silica materials are important for further surface modification, gas adsorption, and other surface phenomena such as wetting. Grafting of functional groups onto the pore walls is also a well-known strategy to design new adsorbents and catalysts [81]. Figure 3 illustrates mesoporous silica materials and their surface characteristics that are important for adsorption applications.

Huang and Yang reported the selective removal of CO_2_ and H_2_S from natural gas using amine-surface-modified silica xerogels and ordered mesoporous silica (MCM-48) [81]. The reported efficiencies towards the removal of CO_2_ and H_2_S were attributed to the high availability of amine groups on the surface of the silica materials. At room temperature, 80% removal of the total amount of CO_2_ (i.e., 50 mg/g of sorbent) was achieved in the first 30 min of the experiment, showing a high adsorption rate and large capacity. Similar results were obtained for the removal of H_2_S, where 80% adsorption was achieved in the first 35 min.

Furthermore, in a number of investigations, the Jones laboratory [91,92,93,94,95,96,97,98,99] demonstrated the utility of amine-modified aluminosilicates for the capture of CO_2_ and other carbonyl compounds including aldehydes and ketones. It was determined that CO_2_ capture is possible through a reversible adsorption of the gaseous molecule onto the aminosilica material. Similarly, the formation of an imine or hemiaminal was required for the capture of aldehydes and ketones. In their analysis of the capacity and recyclability of these materials for CO_2_ remediation, it was determined that CO_2_ adsorbs reversibly and that the material remains stable after 50 cycles of repetitive adsorption–desorption cycling. The capture event exhibits very fast reaction kinetics, reaching up to 90% of the total capacity (up to 7.9 mmol·g^−1^) of the material within the first few minutes of treatment. Consequently, these materials represent a viable alternative to traditional CO_2_ capture by aqueous amines and other silica supported amines in that they are less expensive, easier to synthesize, and exhibit greater performance and stability [97]. Rather than a post-treatment functionalization technique applied to a scaffold material, these materials incorporate the amine functionality during its fabrication by the ring-opening polymerization of aziridine. However, this incorporation limits the use of the material uniquely to contaminants that react with amines, not being possible to easily alternate to other functionalities for the capture of contaminants that may not react with amines. Additionally, the aziridine monomer is somewhat difficult and potentially dangerous to handle at scale without specialized equipment.

The Jones group also used amine-functionalized porous silica as an aldehyde abatement material to capture low-molecular weight aldehydes (e.g., formaldehyde). Specifically they determined that 1.4 mmol·g^−1^ of formaldehyde was retained in silica materials bearing primary amines, 0.8 mmol·g^−1^ for silica bearing secondary amines, and a negligible amount for tertiary amines. These results suggest that the primary and secondary amines are better suited for capturing aldehydes rather than the tertiary amines, consistent with covalent capture of the target contaminant by formation of imine and hemiaminal intermediates. In the same study, they also investigated the capture of a few higher molecular weight, less volatile aldehydes. Unfortunately, the reaction time necessary to achieve equivalent performance with these target contaminants was in excess of 10 h, a much longer period of time compared to formaldehyde adsorption [91]. Therefore, this extended reaction time may impose a limitation on the application of these materials in an industrial environment, where a fast remediation of the contaminant may be fundamentally necessary.

In addition to gaseous capture, silica based materials have been reported for the removal of organic dyes from wastewater. Tsai [78] investigated the functionalization of mesoporous silica with –COOH groups due to the fact that carboxylic acid can form hydrogen bonds with different types of compounds, such as metal ions, dyes, and pollutants. The group was able to successfully functionalize mesoporous silica SBA-16 with tunable loadings of carboxylic acid groups. The study indicated that the specific interactions between the carboxylic acid functional groups and the target adsorbate molecules only occurred at specific pH values, consistent with their proposed hydrogen bonding capture model. For instance, the maximum uptake for methylene blue was obtained at a pH = 9. While these materials may be effective adsorbents under basic conditions, the pH dependency likely imposes a limitation on the practical utility of material. Furthermore, in a recent published review article, Vunain et al. [112] summarized a series of amino-functionalized [102,103,104,105,106] aminopropyl-functionalized [107,113,114,115,116,117] and thiol-functionalized [108,109,110,111,118] silica materials for the remediation of different metal ions, such as Cd^2+^, Co^2+^, Cu^2+^, Zn^2+^, Ni^2+^, Al^2+^, Cr^3+^, Pb^2+^, Hg^2+^, and U^6+^.

## 3. Carbon-Based Nanomaterials

**Figure 4:**
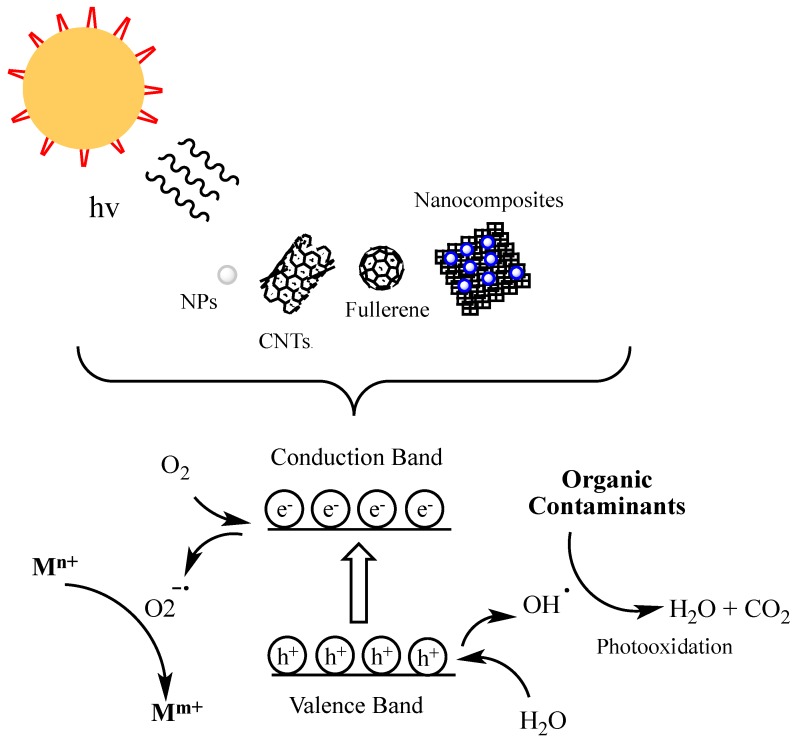
Photocatalytic degradation mechanisms of metal and organic contaminants.

The structural composition of elemental carbon and its mutable hybridization states account for the unique physical, chemical, and electronic properties of carbonaceous materials [119] compared to metal-based nanomaterials. Mutable hybridization states can yield different structural configurations such as fullerene C_60_, fullerene C_540_, single-walled nanotubes, multi-walled nanotubes, and graphene [14]. In a variety of investigations determining the suitability of carbon nanotubes and graphene for environmental remediation applications, it has been reported that surface treatments, activation, or functionalization of the pristine carbon material is first required. Multi-walled and single walled carbon nanotubes (MWCNTs and SWCNTs) have been the subject of many studies. The adsorption properties of these materials make them particularly useful for the removal of organic and inorganic pollutants from air and from large volumes of aqueous solution [119,120,121,122]. Carbon based nanomaterials are also employed to remediate contaminants through photocatalytic approaches. Figure 4 demonstrates the photocatalytic approach for the remediation of environmental contaminants. Under UV irradiation, photons of energy greater than or equal to the band gap of the nanotubes promote the generation of valence band holes (h+) and conduction band electrons (e−). The holes are responsible for the formation of hydroxyl radicals that take part in the oxidation of chlorinated organic compounds. The electrons form superoxide radicals that take part in the reduction of heavy metal contaminants. Several studies have been reported that describe the use of graphene to fabricate photocatalytic nanocomposites [123,124,125,126]. Graphene composites containing TiO_2_ NPs show increased photocatalytic activity when compared to bare TiO_2_ NPs due to an increase in conductivity [123].

### 3.1. Graphene Materials

Both pristine graphene or its modified form have been investigated for environmental remediation applications. Table 3 summarizes some of the different types and its applications in environmental remediation.

Li et al. [127] used pristine graphene as an effective adsorbent for the removal of fluoride from an aqueous solution. The monolayer adsorption capacity of fluoride by graphene was 35.59 mg/g at 298 K and pH = 7.0. The large adsorption capacity and efficiency make graphene a promising fluoride adsorbent. Even though pristine graphene can be used for environmental remediation applications, a variety of techniques rely on the use of modified graphenes for the remediation of different compounds [133]. Surface modifications decrease the aggregation of the graphene layers and thus increase the effective surface area, making modified graphene a more promising material than pristine graphene [134]. Graphene oxide (GO) is an example of modified graphene that has been described for environmental remediation by adsorption of a variety of gaseous and water contaminants, such as SO_x_, H_2_S, NH_3_, volatile organic compounds, heavy metals, pesticides, and pharmaceuticals. Several oxygen-containing functional groups—such as carboxylic acids, epoxides, and hydroxyls—are present on the carbon surface of graphene oxides (GOs) [133]. The strong acidity of the layered GO structure facilitates acid-base interactions with basic contaminant gases, such as ammonia [133]. In this context, Seredych and Bandosz [128] reported the removal of ammonia, monitored by an increase in the pH of the GO material bearing epoxy, OH and COOH on their surfaces which associated with 24.4% water content revealing a pH 3.16. This value increased to 7.66 after NH_3_ adsorption and the water content decreased by 75%. Furthermore, their GO material was reported to adsorb a much higher amount of NH_3_, (i.e., 61 mg of NH_3_/g of GO), than oxidized activated carbon samples (26.7 mg of NH_3_/g of oxidized carbon sample) [128]. In addition, GO has been reported to present high adsorption capacity for cationic metals. Nonetheless, the removal of anionic metals requires the modification of GO with organic or metal oxides [133].

Y. Zhang et al. [123] used TiO_2_-graphene nanocomposites for the remediation of benzene, and evaluated the influence of different ratios of graphene on the photocatalytic activity of the nanocomposite. Although their initial enhancement of the photocatalytic activity was successful with a use of higher ratios of graphene, the results suggest that beyond the optimum graphene ratio, the photocatalytic activity can decrease with further addition of graphene. Consequently, controlling the composition ratio in the nanocomposite is essential for achieving an optimal photocatalytic performance, which for this study was obtained using 0.5 weight % graphene. Other graphene percentages used in the study were 0.2, 1, 2, 5, 10, and 25–30%. For the best formulation, (i.e., 0.5% graphene), the conversion of benzene was maintained at 6.4% for up to 28 h of reaction, indicating that the material has a very stable activity toward the degradation of benzene.

Furthermore, ZnO-graphene and CdS-graphene composites have also shown photocatalytic capabilities towards water contaminants. Liu et al. [124] synthesized ZnO-graphene for the photocatalytic reduction of Cr^6+^ under UV irradiation. The removal rate obtained for the composite was 40% higher than the removal rate obtained for pure ZnO material. Similar to the experiments reported by Y. Zhang et al. [123], Liu and coworkers [124] investigated how different weight percentages of graphene can affect the removal rate of Cr (VI) contaminants in water. As the percentage of graphene increased the removal rate initially increased, reaching a maximum value of 98% for the formulation containing 1.0 wt % of graphene. Nonetheless, further increase in the graphene content resulted in a decrease of the removal rate. In another similar study, N. Zhang et al. [132] reported the effect of different weight addition ratios of graphene in CdS-graphene nanocomposites, also showing that excessive addition of graphene can lower the photoactivity of the nanocomposite due to lower light intensity and decreased amount of CdS (the primary photoactive ingredient of the composite). They indicated that 5% is the optimum weight addition ratio of graphene, which can promote a 100% reduction of Cr^6+^ in water within 20 min. This percent reduction is much higher than the reported value for unadulterated CdS (i.e., 39% reduction). The modification of graphene with other components, such as metal oxides, promotes an enhancement in the material’s applicability as it expands the array of contaminants it can degrade.

### 3.2. Carbon Nanotubes (CNTs)

Most notably, efforts have been undertaken to open the closed ends of pristine CNTs in order to enhance their adsorption properties [135]. Generally, SWCNTs are arranged in a hexagonal configuration (i.e., one nanotube surrounded by six others), thus forming bundles of aligned tubes with a heterogeneous, porous structure. For a typical open-ended CNT bundle, adsorption can take place in four different available sites, which are of two types: those with lower adsorption energy, localized on external surfaces of the external CNTs composing the bundle; and those of higher adsorption energy, localized either in between two neighboring tubes or within an individual tube [119]. Adsorption on external sites reaches equilibrium much faster than adsorption on internal sites due to the direct exposure of the external sites to the adsorbing material. MWCNTs do not usually exist as bundles, except when specific methods of preparation are used to create such configurations. Yang et al. [136] demonstrated in their nitrogen adsorption studies that different types of pores (i.e., inner and aggregated) create a multi-stage adsorption process. Aggregated pores were shown to be more significantly responsible for the adsorption properties of these materials than the less accessible inner pores.

Furthermore, another factor that can influence the adsorption capacity of CNTs is the oxygen content. Depending on the specific synthetic procedures and the associated purification processes, CNTs may contain –OH, –C=O, and –COOH groups that can positively affect adsorption capacities. Different chemicals—such as HNO_3_, KMnO_4_, H_2_O_2_, NaOCl, H_2_SO_4_, KOH, and NaOH—can be used to oxidize CNTs. Increased Pb^2+^, Cd^2+^, Ni^2+^, and Cu^2+^ adsorption capabilities were reported for CNTs oxidized with nitric acid [61]. Furthermore, for pH controlled experiments, Li et al. [137] reported that the amount of cationic dyes adsorbed increased with the pH due to electrostatic attraction between the CNT surface and the positively charged dyes. Although the properties of the CNTs adsorbate play an important role in the efficacy of contaminant adsorption, many physicochemical properties of the adsorbate gas, such as molecular weight, permanent electric dipole moment, and critical temperature can significantly affect the adsorption phenomenon of these CNTs [135].

In conclusion, pristine carbon-based nanomaterials themselves are often inert toward environmental contaminants without modification. In order to improve their efficiency, they typically must be modified or coated with other reactive materials having appropriate functional groups or charges. Thus, these hybrid materials gather multiple features into one template in order to increase the desired performance.

## 4. Polymer-Based Nanomaterials

Although the large surface area-to-volume ratio of nanomaterials contributes to higher reactivity with concomitant improved performance, the occurrence of aggregation, non-specificity, and low stability can limit the use of these nanotechnologies due to the lack of functionality. An alternative to enhance stability of nanoscale materials is to employ the use of a host material, the purpose of which is to serve as a matrix or support to other types of materials (e.g., NPs) [13,138]. Table 4 provides a brief summary of some polymer-based nanomaterials used for remediation of environmental contaminants.

Polymers are mostly used for the detection and removal of contaminant chemicals (e.g., manganese, nitrate, iron, arsenic, heavy metals, etc.), gases (e.g., CO, SO_2_, NO_x_), organic pollutants (e.g., aliphatic and aromatic hydrocarbons, pharmaceuticals, or VOCs) and a wide array of biologics (e.g., bacteria, parasites, viruses, etc.). Polymeric hosts (e.g., surfactants, emulsifiers, stabilizing agents, and surface functionalized ligands) are often employed to enhance stability and overcome some of the limitations of pristine NPs as well as to impart other desirable properties such as enhanced mechanical strength, thermal stability, durability, and recyclability of the material in question.

Amphiphilic polyurethane (APU) NPs have been developed for the remediation of polynuclear aromatic hydrocarbons (PAHs) from soils, thus validating the hypothesis that organic NPs can be engineered with desired properties [139]. The hydrophilic surface of the nanoparticles promotes mobility in the soil, while the hydrophobic interior of the material confers affinity for the hydrophobic organic contaminants. APU NPs removed phenanthrene from contaminated aquifer sand (i.e., 80% recovery). An analysis of different formulations indicated that the APU nanoparticle affinity for phenanthrene increased when the size of the hydrophobic backbone was also increased. Furthermore, increasing the number of ionic groups on the precursor chain contributed to a reduction in APU particle aggregation in the presence of polyvalent cations [139]. While the application of these materials in the environment could be beneficial for contaminant remediation, there is no report on the biodegradability of such materials, which contributes to concerns regarding their fate after application.

Poly (amidoamine) or dendrimers (PAMAM) have been utilized in wastewater remediation for water samples contaminated with metal ions such as Cu^2+^. Those dendritic nanopolymers contain functional groups such as primary amines, carboxylates, and hydroxamates which are able to encapsulate a broad range of solutes in water, including cations (e.g., Cu^2+^, Ag^+^, Au^+^, Fe^2+^,Fe^3+^, Ni^2+^, Zn^2+^, and U^6+^) [140]. They are used as chelating agents and ultrafilters to bind with metal ions thus facilitating water purification. These materials have also been used as antibacterial/antivirus agents [146]. The key feature of the dendritic nanopolymers is that they have a lower tendency to pass through the pores of ultrafiltration membranes compared to linear polymers of similar molecular weight polymers due to their lower polydispersity and globular shape. Therefore, they have been employed to improve ultrafiltration (UF) and microfiltration (MF) processes for the recovery of dissolved ions from aqueous solutions. First, a solution of functionalized dendritic nanopolymers is mixed with contaminated water and then the bound nanopolymer-contaminants is transferred to UF or MF units to recover the clean water. They can be separated from each other by changing the acidity (pH) of the solution and then the recovered concentrated solution of contaminants is collected for disposal and the nanopolymers may be recycled [147].

**Figure 5:**
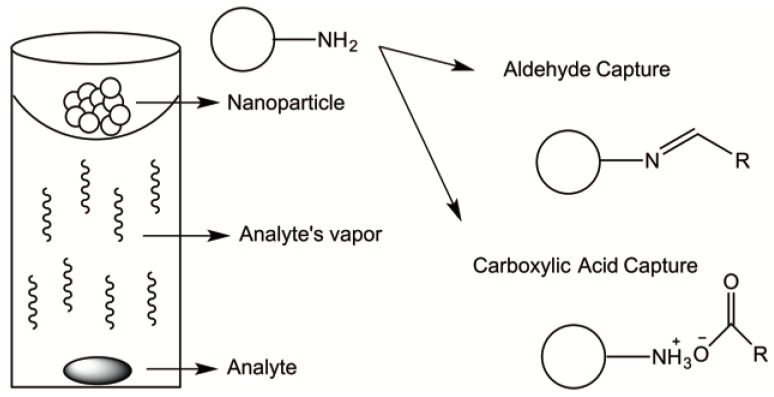
PEI functionalized polymeric nanoparticle for the capture of aldehydes and carboxylic acids.

In a different approach, our group has described the use of functionalized biodegradable and nontoxic polymeric nanomaterials for target-specific capture of VOCs [6,7,141]. Biodegradability is an important and desired feature as it eliminates concerns regarding the fate of the materials after their application. Non-toxicity is another important issue that should be taken into account when designing new environmental remediation technologies. In our work, the incorporation of amine functional groups from poly(ethyleneimine) (PEI) onto the surface of self-assembled PLA-PEG polymeric nanoparticles allowed for the capture of specific VOCs bearing aldehyde and carboxylic acid functional groups by means of condensation (i.e., imine formation) and acid/base reactions, respectively. Thus, the target-specific reactivity of the modified NPs allowed for the rapid and selective capture of desired contaminants in the gas phase (i.e., up to 98% removal). While some technologies rely on physical interactions that could potentially be easily dissociated, this technology is based on the formation of covalent or ionic bonds between the amine functional groups and the targeted aldehyde and/or carboxylic moieties, which may contribute to a more specific and robust capture technology. Figure 5 demonstrates the mechanisms of aldehyde and carboxylic acid gaseous capture.

Through headspace analysis via gas chromatography, we demonstrated that the amine-functionalized NPs were able to significantly reduce aldehydes (by greater than 69% depending on the analyte structure) and carboxylic acid vapors (by greater than 76%). Aldehydes and carboxylic acids of different chain lengths, linear and branched, were efficiently captured by the PEI functionalized nanoparticle. The NPs were also efficient in the capture of aldehyde and carboxylic acids even in the presence of comparable or more volatile non-targeted vapors, thus exhibiting a selective and targeted capture characteristic [7]. In a later iteration of the strategy we successfully modified cellulose nanocrystals (CNCs) with PEI for the efficient capture of aldehyde VOCs [141].

In a similar context, polymer-supported nanocomposites (PNCs) consist of materials that utilize a polymer as a host material on which NPs are either interspersed or coated on top. This material combines the desirable properties of both the polymer host (i.e., exquisite mechanical strength) and the NPs (i.e., high reactivity, arising from their large surface area). Various polymers have also been used in the fabrication of membranes that incorporate metal and metal oxide NPs for environmental remediation applications [17,148,149,150]. In polymeric nanocomposites, polymers are typically used as host materials and other constituents of the composite, such as NPs, are responsible for the contaminant remediation [13]. However, functionalized polymeric nanomaterials have also been described as the main agent responsible for the remediation. Polymer nanomaterials can be used to design technologies exhibiting target-specific capture of compounds from gaseous mixtures in order to prevent off-target fouling that could otherwise contribute to a decrease in the material’s performance [7].

Polymer/inorganic hybrid nanomaterials have also been widely investigated in environmental applications and notably studied for the adsorptive removal of various toxic metal ions, dyes, and microorganisms from water/wastewater streams. They exhibit high stability in terms of chemical and thermal properties. Further, these hybrid materials also demonstrate a high capacity for and selective sorption of heavy metals form aqueous media. They have been fabricated through different strategies such as sol–gel processes, self-assembly techniques, and assembling of nanobuilding blocks, gathering the features of both materials.

Different studies have shown the importance of the integrated hybrid nanoparticles. For example, Khare et al. [142] have designed chitosan-based carbon nanofibers (CNFs) incorporated with iron oxide nanoparticles along with polyvinyl alcohol nanocomposite films. This unique combination of materials revealed an efficient adsorption capacity of Cr^6+^ from water and showed a high capacity for metal uptake (~80 mg /g of chitosan/iron-CNF composite). Mittal et al. [143] have reported that impregnation of SiO_2_ NPs in acrylamide hydrogel improves the monolayer adsorption capacity of acrylamide hydrogel to remove 1408.67 mg/g of cationic dyes (MB) (efficiency = 96%) with the hydrogel nanocomposite dose of 0.2 g/L. They also fabricated SiO_2_ NPs and gum karaya grafted with poly (acrylic acid-co-acrylamide) nanocomposite containing hydrogel for removal of methylene blue (MB) by adsorption from aqueous solutions. These data demonstrated that the grafted copolymerized gum karaya hydrogel could be used as an eco-friendly and efficient adsorbent for the removal of methylene blue dye from industrial wastewater.

Two of the key elements for the use of polymeric nanocomposites are the biocompatibility and biodegradability. For instance, Sun et al. [144] have developed green hybrid nanomaterial adsorbent constituted of wheat xylan/poly(acrylic acid) NP hydrogel encapsulating Fe_3_O_4_ nanoparticles. With this formulation, they achieved 90% removal of methylene blue dye due to the behavior of the magnetic hydrogel material. Similarly, high adsorption capacity of Ni^2+^, Co^2+^, Pb^2+^, Cd^2+^, Cu^2+^, and Cr^2+^ ions from aqueous solution were realized by formulating a nanogel composed of sodium alginate loaded with tetrasodium thiacalixarene tetrasulfonate and Fe_3_O_4_ [151].

Another Fe_3_O_4_ magnetic NP formulation modified with a combination of 3-aminopropyltriethoxysilane and (acrylic/crotonic acid) copolymers was prepared by Ge et al. [145]. The resultant hybrid NPs (diameter ~15–20 nm) were then applied for the removal of Cu^2+^, Cd^2+^, Pb^2+^, and Zn^2+^ from metal contaminated water. It was found that when AgNPs are embedded in cellulose acetate fibers, the resulting material affords potent antibacterial activity [152]. When the same group imbedded AgNPs and Ag^+^ into a mixture of polymethoxybenzyl and poly (l-lactic acid)-*co*-poly (3-caprolactone) nanofibers, the materials revealed antimicrobial properties against *Escherichia coli*, *Staphylococcus aureus*, *Aspergillus niger*, and *Salmonella enterica* [24,153,154,155]. Further, dispersing AgNPs (1–70 nm) into polysulfone membranes does not alter the membrane structure. The impregnation of only 0.9 wt % of AgNPs to the NCs membrane caused a 99% decrease in the number of *E. coli* bacteria that were able to grow on the membrane surface. Moreover, these AgNPs were able to reduce the attachment of an *E. coli* suspension to the surface of the immersed membrane by 94% [156].

It is quite clear that both polymer NPs and polymer NCs play a critical role in environmental remediation thanks to their unique physicochemical characteristics which enable them to efficiently remove several contaminants following different mechanisms.

Below in Table 5 we present miscellaneous nanoparticle models that demonstrate efficiencient removal of some pollutants for environmental applications.

## 5. Conclusions

Inorganic, carbonaceous, and polymeric nanomaterials are among the different types of materials that can be successfully employed for a variety of environmental remediation applications. Selecting the best nanomaterial to mitigate a particular pollutant in a specific environmental context requires a full analysis of the type of contaminant to be removed, the accessibility to the remediation site, the amount of material needed to implement efficient remediation, and whether it is advantageous to recover the remediation nanomaterial (recycling). In that each material has its own advantages and issues related to its applicability, we provided here an overall perspective of some nanomaterials that have been utilized in the context of environmental remediation.

Although a variety of studies have been undertaken to investigate the use of nanotechnology, concerns regarding the application of nanotechnology for environmental remediation purposes have yet to be addressed. Also while many studies do demonstrate efficacy in laboratory settings, more research is necessary in order to fully understand how nanotechnology can significantly affect the remediation of environmental contaminants in real case scenarios (e.g., the remediation of contaminated water, soil, and air from industrial processes). Also, while the mechanisms through which the different nanotechnologies are applied are well known, what happens to these materials after they have been applied for contaminant capture or degradation is underexplored. Even though the recyclability of some materials have been described, it appears that at some point the efficacy of these materials declines, which makes them no longer useful. Therefore, research is necessary to elucidate the fate of these materials after introduction to the environment for remediation purposes in order to avoid the possibility of these materials becoming themselves a source of environmental contamination.

These challenges must be overcome to realize the full potential of nanomaterials for environmental applications. Nevertheless, nanotechnology provides a wealth of strategies that can be leveraged to address environmental contamination.

## Figures and Tables

**Table 1:** Metal-based nanomaterials and applications in environmental remediation of contaminants.

Material	Application	Reference
Ag NPs/Ag ions	Water disinfectant—*E. coli*	[20,21,22,23,24]
TiO_2_ NPs	Water disinfectant, soil—MS-2 phage, *E. coli*, hepatitis B virus, aromatic hydrocarbons, biological nitrogen, phenanthrene	[25,26,27,28,29]
Metal-doped TiO_2_	Water contaminants—2-chlorophenol, endotoxin, *E. coli*, Rhodamine B, *Staphylococcus aureus*	[30,31,32,33,34]
Titanate nanotubes	Gaseous—Nitric oxide	[35]
Binary mixed oxide	Water—Methylene blue dye	[36]
Iron-based	Water—Heavy metals, chlorinated organic solvents	[37,38,39,40,41,42,43]
Bimetallic NPs	Water, soil—Chlorinated and brominated contaminants	[44,45,46,47,48,49,50,51,52,53]

**Table 2:** Silica nanomaterials and applications in environmental remediation of contaminants

Material	Application	Reference
Amine-modified xerogels	Gaseous—CO_2_, H_2_S	[81]
Amine-modified aluminosilicates and porous silica	Gaseous—CO_2_, aldehydes, ketones	[91,92,93,94,95,96,97,98,99]
Carboxylic acid-functionalized mesoporous silica	Wastewater—Cationic dyes, heavy metals	[78,100,101]
Amino-functionalized mesoporous silica	Wastewater—Heavy metals	[102,103,104,105,106,107]
Thiol-functionalized mesoporous silica	Wastewater—Heavy metals	[108,109,110,111]

**Table 3:** Graphene materials and their use in environmental remediation

Material	Application	Reference
Pristine graphene	Water—Fluoride	[127]
Graphene oxide	Water/Gaseous—SO_x_, H_2_, NH_3_, heavy metals, pesticides, pharmaceuticals	[31,128,129,130,131]
ZnO-graphene/CdS-graphene	Water—Heavy metals	[132]
TiO_2_-graphene	Gaseous—Benzene	[123,126]

**Table 4:** Polymer-based materials for environmental remediation of contaminants

Material	Application	Reference
Amphiphilic polyurethane NPs	Soil—Polynuclear aromatic hydrocarbons	[139]
PAMAM dendrimers	Wastewater—Heavy metals	[140]
Amine-modified PDLLA-PEG	Gaseous—VOCs	[6,7]
Polyamine-modified Cellulose	Gaseous—VOCs	[141]
Polymer nanocomposites (PNCs)	Water—Metal ions, dyes, microorganisms	[142,143,144,145]

**Table 5:** Lists of some examples of additional nanomaterials for environmental application.

Type of Nanoparticles	Removal Target	Reference
Ag-doped TiO_2_	2,4,6-Trichlorophenol	[157]
Ag-doped TiO_2_ nanofibers	Methylene blue dye	[158]
Cu/Fe/Ag-doped TiO_2_	Nitrate (NO_3_^−^)	[159]
Silica nanoparticles prepared by mixing salicylic acid and hyper-branched poly (propylene imine)	Removal of polycyclic aromatic hydrocarbons (PAH), such as pyrene and phenanthrene, and Pb^2+^, Hg^2+^, Cd^2+^, Cr_2_O_7_^2−^ from contaminated aqueous solutions	[160]
PAMAM dendrimer composite membrane consisting of chitosan and a dendrimer	Separation of CO_2_ from a feed gas mixture of CO_2_ and N_2_ on porous substrates	[161]
Fe^0^ coated with carboxymethyl cellulose polymer matrix	Hexavalent chromium (Cr^6+^) from aqueous solutions	[162]
Gold coated with chitosan polymer	Zn^2+^, Cu^2+^ form aqueous solutions	[163]
Poly (methacrylic acid)-grafted chitosan/bentonite	Th^4+^	[164]
Carbon nanotubes/Al_2_O_3_ nanocomposite	Fluoride	[165]
Multiwall carbon nanotube (MWCTs)	Zn^2+^	[166]
